# Acute kidney injury in multiple myeloma patients undergoing autologous hematopoietic stem cell transplant: a cohort study

**DOI:** 10.1007/s40620-023-01809-3

**Published:** 2023-11-29

**Authors:** Natacha Rodrigues, Claudia Costa, Carolina Branco, Carlos Martins, José António Lopes

**Affiliations:** 1 Division of Nephrology and Renal Transplantation, Centro Hospitalar Universitário Lisboa Norte, EPE, Avenida Professor Egas Moniz, 1649-035 Lisbon, Portugal; 2 Division of Haematology, Centro Hospitalar Universitário Lisboa Norte, EPE, Lisbon, Portugal

**Keywords:** Acute kidney injury, Multiple myeloma, Autologous hematopoietic stem cell transplant, Epidemiology

## Abstract

**Background:**

Autologous hematopoietic stem cell transplant plays an important role in multiple myeloma (MM) treatment. Increasing incidence of MM and growing awareness of acute kidney injury (AKI) as a complication of hematopoietic stem cell transplant results in the need to better understand AKI in these patients. We aimed to evaluate incidence, risk factors and 5-year prognostic impact of AKI in MM patients undergoing autologous hematopoietic stem cell transplant.

**Methods:**

Retrospective cohort study. AKI was defined by the KDIGO classification using creatinine and urinary output criteria. We used survival analysis methods considering competing events for risk factors and disease-free survival, Cox proportional regression for overall survival and stepwise regression methods for multivariable models.

**Results:**

We analyzed data regarding 143 patients. The cumulative incidence of AKI and moderate-to-severe AKI was 49.7% and 14.1%, respectively. Factors with independent impact on AKI were obesity (HR: 1.83, 95% CI 1.07–3.11; *p* = 0.026), Hematopoietic cell transplantation—specific comorbidity index (HCT-CI) ≥ 2 (HR: 1.85, 95% CI 1.08–3.17), chronic kidney disease (CKD) (HR: 2.06, 95% CI 1.05–4.04), amyloidosis (HR: 2.25, 95% CI 1.25–4.06), mucositis grade 3–4 (HR: 2.19, 95% CI 1.25–3.86) and exposure to nephrotoxic drugs (HR: 2.0856, 95% CI 1.04–4.19). Moderate-to-severe AKI had an impact (HR: 1.62, 95% CI 1.15–2.31) on 5-year overall survival.

**Conclusion:**

Acute kidney injury affects almost half of MM patients undergoing autologous hematopoietic stem cell transplantation, and reduction in urinary output allows early diagnosis in almost a quarter of the patients. Obesity, HCT-CI ≥ 2, CKD, amyloidosis, mucositis grade 3–4 and exposure to nephrotoxic drugs are significant risk factors. Moderate-to-severe AKI is associated with lower 5-year overall survival.

**Graphical abstract:**

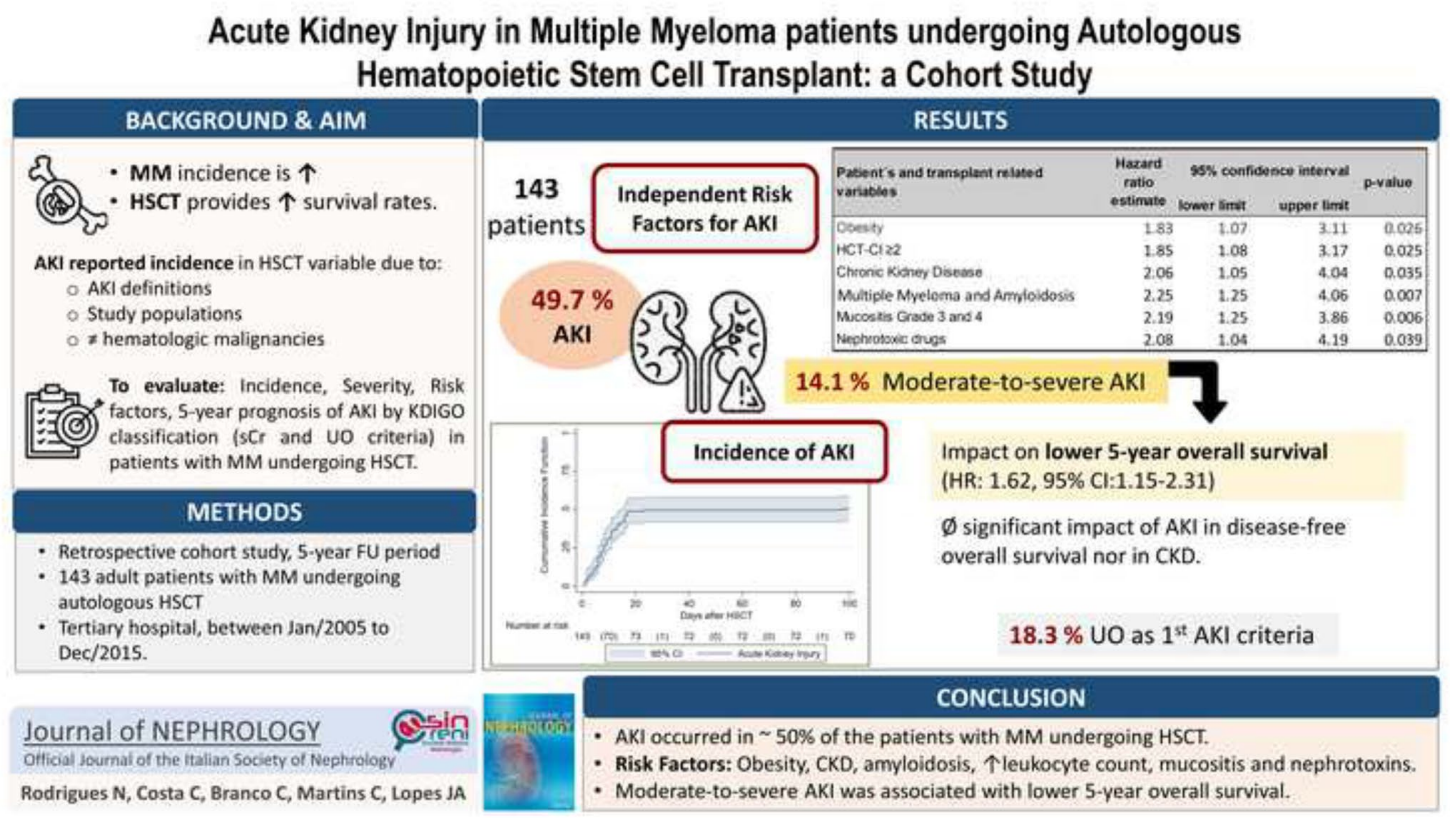

## Introduction

Multiple myeloma is the second most common hematological malignancy after non‐Hodgkin lymphoma and its incidence has been rising around the globe [1]. Treatment of this monoclonal gammopathy has evolved in the past two decades resulting in an overall decrease in mortality [2], and autologous hematopoietic stem-cell transplant (HSCT) remains an important part of the treatment of these patients as consolidation therapy after completing conditioning regimen with high-dose chemotherapy [3]. The awareness that a growing number of patients with multiple myeloma are undergoing autologous hematopoietic stem-cell transplant highlights the need to better characterize and study the prognostic impact of complications associated with this procedure and to analyze them separately from patients undergoing hematopoietic stem-cell transplant for other clinical conditions. Acute Kidney Injury (AKI) occurring in the first 100 days is a known complication of this procedure and is receiving increasing attention because of the clinical impact of AKI on patients' outcomes [4,5].

The heterogeneity of AKI definitions and the inclusion of patients with different hematologic diagnoses in the studies in the early 2000s resulted in a wide range of reported incidence in HSCT. In 2012, the Kidney Disease Improving Global Outcomes (KDIGO) classification for AKI [6] led to an agreed AKI definition, which includes an increase in serum creatinine of at least ≥ 0.3 mg/dL or ≥ 50% within 48 h, or urinary output of < 0.5 mL/kg/hour for at least 6 h.

In the last five years, the incidence of  AKI, defined according to the KDIGO classification has been reported in allogeneic HSCT mainly in patients with leukemia; incidence varied from 55.1% to 68.9% with a negative impact on survival [7–11]. Acute kidney injury in autologous HSCT has been less studied with the KDIGO classification. One study in patients with lymphoma reports an incidence of 63.7% with moderate to severe AKI associated with lower overall survival [12], and one study in patients with multiple myeloma reports a 10.3% incidence of AKI calculated by serum creatinine criteria alone [13].

Our study aims to define the incidence and severity of AKI in patients with multiple myeloma undergoing autologous HSCT using the KDIGO classification with both serum creatinine and urinary output criteria, to identify independent risk factors for AKI in these patients and to evaluate the impact of AKI on overall survival, on disease-free survival, on chronic kidney disease (CKD) development and on estimated glomerular filtration rate (eGFR) reduction in the first 5 years after HSCT.

## Materials and methods

We performed a single-center retrospective cohort study including all patients with multiple myeloma undergoing autologous HSCT at the Centro Hospitalar Universitário Lisboa Norte, EPE (CHULN, EPE) between January 2005 and December 2015. We excluded patients under the age of 18 years, patients who had received a previous HSCT, patients with chronic kidney disease already on renal replacement therapy, and patients who underwent renal replacement therapy within one week before HSCT.

Chemotherapy regimens before transplant included the following drug combinations: cyclophosphamide, bortezomib and dexamethasone; bortezomib, doxorubicin and dexamethasone; vincristine, doxorubicin and dexamethasone; doxorubicin, thalidomide and dexamethasone; thalidomide and dexamethasone; lenalidomide and dexamethasone.

Cyclophosphamide (3 g/m^2^) and Granulocyte colony-stimulating factor (10 to 16 μg/kg) were used to mobilize hematopoietic stem cells which were then collected from bone marrow or peripheral blood. Melphalan (dose 140 or 200 mg/m^2^ administered in single dose or divided in two days) was used as conditioning regimen and all patients received ciprofloxacin, acyclovir and fluconazole as prophylaxis.

We collected data from the patients’ medical records in our institution from diagnosis until five years after HSCT. We collected demographic variables (age, gender, race, body mass index (BMI), comorbidity variables (diabetes mellitus, hypertension, CKD, arrythmia, valvular heart disease, ischemic heart disease, cerebrovascular disease, chronic liver disease, intestinal inflammatory disease, peptic ulcer, connective tissue disease, chronic obstructive pulmonary disease, solid-organ cancer, psychiatric disease), multiple myeloma-related variables (subtype, light chain domain, concomitant amyloidosis, International Staging System classification, cytogenetic abnormalities, bone marrow plasma cells percentage, M-protein level, Serum B2-microglobulin level, number of previous lines of therapy, number of chemotherapy cycles, radiotherapy in the past), HSCT-related variables (blood results at hospital admission for HSCT, graft source, period of aplasia, sepsis, fever, shock, nephrotoxic drugs, mucositis, sinusoidal obstructive syndrome, thrombotic microangiopathy, tumor lysis syndrome, AKI, AKI stage).

### Definitions

Baseline eGFR was estimated according to the CKD-EPI equation [14] considering serum creatinine at the last medical appointment before HSCT.

Acute kidney injury diagnosis was made based on daily values of serum creatinine and 6-h urinary output from hospital admission for HSCT until hospital discharge, and on all hospital admissions and weekly evaluations in the outpatient clinic in the first 100 days after HSCT. Acute kidney injury was defined by KDIGO criteria [6] (any of the following: increase in serum creatinine by ≥ 0.3 mg/dl (≥ 26.5 µmol/l) within 48 h; or increase in serum creatinine to ≥ 1.5 times baseline, which is known or presumed to have occurred within the prior 7 days; or urinary output < 0.5 ml/kg/h for 6 h). Stage of AKI followed KDIGO classification considering the worst serum creatinine value and/or longest period of urinary volume reduction. Moderate to severe AKI was defined as AKI stage 2 and AKI stage 3.

Chronic kidney disease stage 3 or higher was defined according to the KDIGO definition [15] as a persistent decrease in eGFR to below 60 mL/min/1.73 m^2^.

The diagnosis of multiple myeloma was made according the International Multiple Myeloma Working Group (IMWG) Criteria [16]. Multiple myeloma staging was made using International Staging System for Myeloma (ISS) [17]. The associated‐amyloid light chain amyloidosis was diagnosed by demonstration of Congo red staining (kidney or subcutaneous fat biopsy). The hematopoietic cell transplantation—specific comorbidity index (HCT-CI) was calculated according to the validated version [18] considering the patient's comorbidities.

Obesity was defined as a BMI above 30 kg/m^2^.

Fever was defined as a measured body temperature of 38 °C (100.4 °F) or greater. Sepsis was diagnosed when patients presented with temperature ≥ 38 °C or < 36 °C, a white blood cell count > 10,000/mm^3^ or < 4000/mm^3^, and a positive blood culture for bacteria [19]. Shock was considered when patients presented with cardiac frequency > 90 bpm, systolic blood pressure < 90 mmHg and at least one lactate determination > 2 mmol/L or 22 mg/dL. Oral mucositis was graded according to the World Health Organization’s (WHO’s) Oral Toxicity Scale [20].

Nephrotoxic drugs considered in this report included gentamicin, amikacin, vancomycin, amphotericin B, and foscarnet.

### Ethical issues

The ethical committee of Centro Académico de Medicina de Lisboa approved this study (approval number 35337) according to institutional guidelines and waived the requirement to obtain informed consent due to its retrospective and non-interventional nature.

### Statistical methods

We present categorical variables as frequencies and continuous variables as median and interquartile range (P25 = 25th percentile; P75 = 75th percentile).

Cumulative incidence of AKI, univariable and multivariable analyses of factors predicting AKI were calculated using the Fine and Gray method [21] as survival analysis method considering competing events, and death was considered a competing risk event. The final multivariable model was created using backward stepwise regression.

In order to evaluate the prognostic impact of AKI, we considered type 1 right censoring for a period of five years after HSCT. The impact of AKI on relapse-free survival was calculated using the Fine and Gray method [21] and death was considered a competing risk event. The final multivariable model was created using backward stepwise regression.

The impact of AKI on overall survival was calculated using a Cox regression model including demographic variables and other variables with level of significance *α* < 0.2 in univariable analysis to create the final multivariable model. The Cox proportional hazards assumption was checked using formal statistical tests and graphical diagnostics based on the scaled Schoenfeld residuals.

The incidence of CKD and eGFR reduction > 25% was calculated at the end of the first year and at the end of 5 years, and the association with AKI in the first 100 days post-HSCT was evaluated.

Our approach followed the European Group for Blood and Marrow Transplantation guidelines on statistical methodology [22]. A level of significance *α* = 0.05 was considered for statistical significance. The statistical software package STATA 16.0 for Windows and R software (R Core Team (2017). R: A language and environment for statistical computing. R Foundation for Statistical Computing, Vienna, Austria. URL https://www.R-project.org/.) were used for the data analysis.

## Results

One hundred and eighty-two patients with multiple myeloma underwent autologous HSCT during the referred period. Thirty-nine patients presented at least one exclusion criteria. One hundred and forty-three patients were included in our final analysis. Table 1 shows the main demographic variables, multiple myeloma-related variables and transplant-related variables.

**Table 1:** Patient's baseline characteristics, multiple myeloma characterization and transplant-related variables

Patient’s characteristics and comorbidities	Category	*n* (%)	Median (IQR)
Age at transplant (years)			59.2 (50.5–63.6)
Gender	Male	88 (61.5)	
Race	Caucasian	130 (90.9)	
	Non Caucasian	13 (9.1)	
BMI (Kg/m^2^)			26.1 (23.4–29.4)
Obesity (BMI > 30 kg/m^2^)		31 (21.7)	
HCT-CI score	0–1	120 (83.9)	
	≥ 2	23 (16.1)	
Diabetes mellitus		18 (12.6)	
Hypertension		53 (37.1)	
Heart failure		10 (7)	
Baseline eGFR (ml/min/1.73 m^2^)			100.4 (89.5—110.8)
Chronic kidney disease	eGFR < 60 ml/min/1.73 m^2^	15 (10.5))	
Multiple myeloma presentation characteristics			
Multiple myeloma	IgG	90 (62.9)	
	IgA	21 (14.7)	
	IgD	3 (2.1)	
	Free light chain kappa	17 (11.9)	
	Free light chain lambda	12 (8.4)	
Light chain domain	Kappa	87	
	Lambda	51	
Multiple myeloma and amyloidosis		5 (3.5)	
ISS	Stage I	70 (49.0)	
	Stage II	38 (26.6)	
	Stage III	35 (24.5)	
Cytogenetic abnormalities		49 (34.3)	
Bone marrow plasma cells (%)			30 (17–67)
M-protein level (gr/dl)			3.3 (3.2–6.6)
Serum B2-microglobulin (mg/L)			3.1 (1.7–5.5)
Serum Ig (mg/dl)			5880 (3490–9200)
Light chain (mg/dl)			3560 (890–7800)
Serum K/L			4.52 (0.26–56.35)
Treatment previous to HSCT			
Nr of previous lines of therapy			1 (1–2)
Nr of chemotherapy cycles			4 (3–4)
Radiotherapy in the past		49 (34.5)	
Auto-HSCT characteristics and complications			
Graft source	Peripheral blood	136 (95.1)	
	Bone marrow	7 (4.9)	
Melphalan	1 day	15 (10.6)	
	2 days	126 (89.4)	
Period of aplasia (days)			11 (11–12)
Sepsis		38 (26.6)	
Fever		111 (77.6)	
Nephrotoxic drugs		106 (74.1)	
Mucositis	Grade 1–4	80 (55.9)	
	Grade 3–4	16 (11.2)	
TMA/TLS/VOD		3 (2.1)	
Shock		4 (2.8)	
At hospital admission			
Hemoglobin (gr/dl)			11.7 (10.8–12.4)
Leukocytes (cells/mm^3^)			5450 (4120–6610)
Neutrophils (cells/mm^3^)			3375 (2330–4765)
Lymphocytes (cells/mm^3^)			1066 (760–1500)
Platelets (/μl)			198,000 (151,000–270,000)
Urea (mg/dl)			33 (28–44)
Uric acid (mg/dl)			5 (4.1–6)
Calcium (mg/dl)			9.1 (8.7–9.4)
Phosphate (mg/dl)			3.4 (3–3.9)
C-Reactive protein (mg/dl)			0.25 (0.09–0.67)
Lactate dehydrogenase (U/L)			333 (297–390)
Albumin (g/dL)			3.9 (3.7–4.2)
Alanine transaminase (U/L)			18 (14–27)
Total bilirubin (mg/dl)			0.5 (0.4–0.6)
Serum B2-microglobulin (mg/L)			2.4 (1.6–3.1)

*IQR* Interquartile range; *BMI* body mass index; *HCT-CI* hematopoietic stem cell transplant comorbidity index; *Nr* number; *eGFR* estimated glomerular filtration rate; *Ig* immunoglobulin; *ISS* international staging system; *TMA/TLS/TMA/TLS/VOD* thrombotic microangiopathy, tumor lysis syndrome or veno-occlusive disease

### Acute kidney injury

**Fig. 1:**
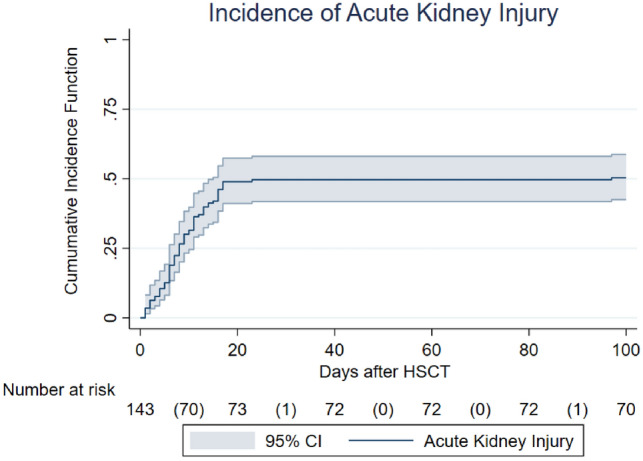
AKI cumulative incidence function considering death as a competing event

The AKI cumulative incidence in the first 30 days and 100 days after HSCT was 47.8% and 49.7%, respectively. AKI occurred within a median time of 8 (5–13) days after HSCT Fig. 1. Moderate-to-severe AKI cumulative incidence was 14.1%.

Considering patients with AKI, the earlier diagnostic criteria for AKI was: serum creatinine elevation in 71.8%; urinary output reduction in 18.3% and both serum creatinine elevation and urinary output reduction in 9.9%.

At presentation, the severity of AKI was stage 1 in 85.9%, stage 2 in 5.6% and stage 3 in 8.5%. The highest severity stage observed was stage 1 in 71.8%, stage 2 in 15.5% and stage 3 in 12.7%. Two patients (1.4%) needed renal replacement therapy. Acute kidney injury total recovery was seen in 72.6% of cases.

Acute kidney injury occurred during the hospital admission period for HSCT in 89.4% of patients.

### Factors correlated with acute kidney injury risk 

In the univariable analysis considering death as a competing risk (Table 2), variables associated with AKI were HCT-CI score ≥ 2 (HR: 2.06, 95% CI 1.24–3.43; *p* = 0.005), baseline eGFR (HR: 0.98, 95% CI 0.97–0.99; *p* < 0.001), CKD (HR: 3.3, 95% CI 1.62–6.71; *p* = 0.001), ISS (HR: 1.55, 95% CI 1.18–2.04; *p* = 0.002), fever (HR: 2.01, 95% CI 1.02–3.97; *p* = 0.044), sepsis (HR: 1.78, 95% CI 1.11–2.85; *p* = 0.016), nephrotoxic drugs (HR: 2.56, 95% CI 1.30–5.06; *p* = 0.007), mucositis (HR: 1.91, 95% CI 1.16–3.14; *p* = 0.001), mucositis grades 3 and 4 (HR: 2.94, 95% CI 1.75–4.92; *p* < 0.001).

**Table 2:** Competitive risk univariable regression analysis for AKI

Patient’s characteristics and comorbidities	Hazard ratio estimate	95% confidence interval		*p*-value
		Lower limit	Upper limit	
Age at transplant (years)	1.02	1.00	1.05	0.057
Gender (reference female)	1.24	0.77	1.99	0.379
Race (reference non Caucasian)	1.18	0.54	2.57	0.684
Obesity	1.64	0.99	2.71	0.054
HCT-CI (reference score < 2)	2.06	1.24	3.43	0.005
Diabetes mellitus	1.57	0.88	2.79	0.120
Hypertension	1.19	0.75	1.90	0.462
Heart failure	1.63	0.80	3.31	0.175
Chronic kidney disease	3.3	1.62	6.71	0.001
Multiple myeloma characteristics and previous treatment				
Light chain domain (reference lambda)	1.18	0.72	1.92	0.508
Multiple myeloma and amyloidosis	2.12	0.92	4.94	0.078
ISS	1.55	1.18	2.04	0.002
Cytogenetic abnormalities	1.56	0.99	2.46	0.055
Bone marrow plasma cells (%)	1.00	0.99	1.01	0.96
M-protein level (gr/dl)	0.99	0.94	1.03	0.609
Serum B2-microglobulin (mg/L)	1.04	1.00	1.08	0.114
Nr of previous lines of therapy	1.25	0.82	1.92	0.288
Nr of chemotherapy cycles	0.92	0.77	1.1	0.375
Radiotherapy in the past	1.18	0.74	1.87	0.484
HSCT characteristics and complications				
Graft source	1.24	0.40	3.88	0.711
Melphalan (reference 1 day)	1.26	0.63	2.54	0.513
Period of aplasia (days)	1.03	0.95	1.1	0.500
Sepsis	1.78	1.11	2.85	0.016
Fever	2.01	1.02	3.97	0.044
Nephrotoxic drugs	2.56	1.30	5.06	0.007
Mucositis 1–4	1.91	1.16	3.14	0.010
Mucositis 3–4	2.94	1.75	4.92	< 0.001
TMA/TLS/VOD	0.69	0.73	6.43	0.741
Shock	2.61	0.69	9.85	0.157

*HCT-CI* Hematopoietic stem cell transplant comorbidity index; *eGFR* estimated glomerular filtration rate; *ISS* international staging system; *Nr* number; *TMA/TLS/VOD* thrombotic microangiopathy, tumor lysis syndrome or veno-occlusive disease

Variables independently associated with a higher incidence of AKI are shown in Table 3 and include obesity (HR: 1.83, 95% CI 1.07–3.11; *p* = 0.026), HCT-CI score ≥ 2 (HR: 1.85, 95% CI 1.08–3.17; *p* = 0.025), CKD (HR: 2.06, 95% CI 1.05–4.04; *p* = 0.035), multiple myeloma and amyloidosis (HR: 2.25, 95% CI 1.25–4.06; *p* = 0.007), mucositis grade 3 and 4 (HR: 2.19, 95% CI 1.25–3.86; *p* = 0.006) and nephrotoxic drugs (HR: 2.0856, 95% CI 1.04–4.19; *p* = 0.039) Table 3.

**Table 3:** Competing risks multivariable regression analysis for AKI

Patient’s and transplant-related variables	Hazard ratio estimate	95% confidence interval		*p*-value
		Lower limit	Upper limit	
Obesity	1.83	1.07	3.11	0.026
HCT-CI ≥ 2	1.85	1.08	3.17	0.025
Chronic kidney disease	2.06	1.05	4.04	0.035
Multiple myeloma and amyloidosis	2.25	1.25	4.06	0.007
Mucositis grade 3 and 4	2.19	1.25	3.86	0.006
Nephrotoxic drugs	2.08	1.04	4.19	0.039

*HCT-CI* Hematopoietic stem cell transplant comorbidity index

### Overall survival and relapse-free survival

In the first year after HSCT, 14 (9.7%) patients died. By the end of the 5-year follow-up period, 51 (35.7%) patients had died.

**Fig. 2:**
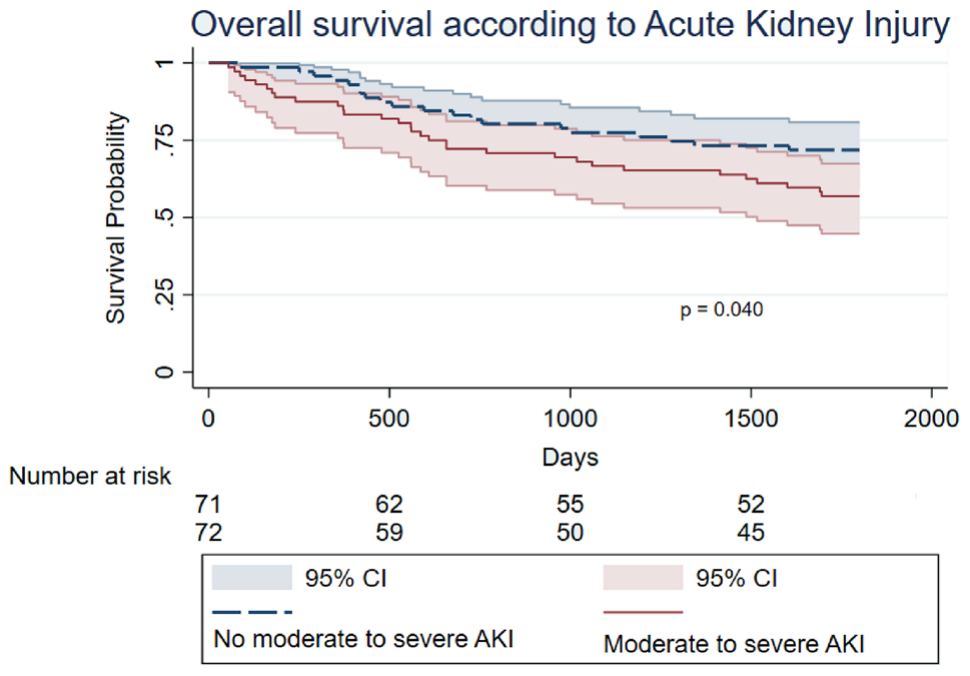
Overall survival in days according to moderate to severe AKI

In univariable analysis, variables with an impact on lower overall survival were moderate to severe AKI (HR: 1.95; 95% CI 1.26–2.98; *p* = 0.040) (Fig. 2), ISS (HR: 1.69; 95% CI 1.22–2.35; *p* = 0.002), cytogenetic abnormalities (HR: 2.21; 95% CI 1.27–3.83; *p* = 0.005) and relapse (HR: 1.25; 95% CI 0.71–2.19; *p* = 0.043).

In our multivariable model, variables with an independent impact on lower overall survival were diabetes mellitus (HR: 3.29.76, 95% CI 1.01–9.72), relapse (HR: 3.05, 95% CI 1.12–8.31), cytogenetic abnormalities (HR: 2.36, 95% CI 1.01–5.53), moderate to severe AKI (HR: 1.62, 95% CI 1.15–2.31) and BMI (HR: 1.10, 95% CI 1.00–1.21) Table 4.

**Table 4:** Multivariable Cox regression for mortality

Variables	Hazard ratio estimate	95% confidence interval		*p*-value
		Lower limit	Upper limit	
Age at transplant (years)	0.98	0.94	1.04	0.711
Gender (reference female)	0.52	0.20	1.30	0.160
BMI (Kg/m^2^)	1.10	1.00	1.21	0.045
Diabetes mellitus	3.29	1.01	9.72	0.048
Hypertension	1.04	0.97	1.02	0.807
Moderate to severe AKI	1.62	1.15	2.31	0.045
ISS stage	1.67	0.89	3.14	0.113
Cytogenetic abnormalities	2.36	1.01	5.53	0.049
Serum B2-microglobulin at diagnosis (mg/L)	1.02	0.95	1.09	0.564
Relapse	3.05	1.12	8.31	0.029

*BMI* Body mass index; *AKI* acute kidney injury; *ISS* international staging system

The cumulative relapse incidence was 61.8% five years after HSCT. The median disease-free overall survival was 43.6 months. No statistically significant association was found between AKI (neither moderate-to-severe AKI nor severe AKI) with lower disease-free overall survival.

### CKD and loss of renal function

The median eGFR prior to HSCT was 100.4 (89.5–110.8) mL/min/1.73 m^2^ and 10.5% of patients had CKD (defined as eGFR<60 mL/min).

At the end of the first year, the median eGFR was 88.6 (68.6–103.3) mL/min/1.73 m^2^, an eGFR reduction > 25% was verified in 18.4% of the patients and CKD prevalence was 17.5%. At the end of the five years, the median eGFR was 82. 6 (60.5–94.8) mL/min/1.73 m^2^, an eGFR reduction > 25% was observed in 32.1% of the patients and CKD prevalence was 27.5%.

No statistically significant association was found between AKI in the first 100 days after HSCT and eGFR reduction > 25% (*p* = 0.819) or CKD prevalence (*p* = 0.101).

## Discussion

In this study, we analyzed the incidence and variables related to AKI in patients with multiple myeloma undergoing autologous HSCT and the impact of AKI on overall survival, relapse-free survival and chronic kidney disease. We did so by considering the most updated AKI definition—the KDIGO classification [6]—using both serum creatinine criteria and urinary output criteria.

We found a cumulative incidence of AKI of 49.7% and of moderate-to-severe AKI of 14.1%. Similar studies on AKI in autologous HSCT reported various incidence rates: Caliskan et al. reported an incidence of 52% [23] in patients with several hemato-oncologic diagnoses, Fadia et al. reported an incidence of moderate-to-severe AKI of 21% [24] in patients with amyloid light chain amyloidosis, Merouani et al. of 56% [25] in patients with breast cancer, Rodrigues et al. of 63.7% [12] in patients with lymphoma and Andronesi of 10.4% [13] in patients with multiple myeloma.

The wide range of these results reflects different AKI definitions in the older studies, omission of the urinary output criteria in the most recent studies and the heterogeneity of baseline diseases.

In our study, AKI urinary output reduction allowed for a first diagnosis of AKI in 18.3% of cases. This finding highlights the importance of monitoring urinary output in these patients and should be taken into account in clinical practice to allow prompt identification and treatment. 

Based on our results, the most important factors associated with AKI are obesity, an HCT-CI score ≥ 2, chronic kidney disease prior to HSCT, the presence of amyloidosis, developing mucositis grade 3 and 4, and exposure to nephrotoxic drugs.

In our study, obesity was associated with a twofold increase in risk for AKI. Obesity has been reported as a risk factor for AKI in other clinical scenarios by Gameiro et al. [26] and Billings et al. [27] in critically ill and septic patients and in patients undergoing cardiac surgery, respectively. The association between BMI and AKI has been explained by the production of inflammatory mediators (adipokines, leptin) by the adipose tissue and by the decreased production of adiponectin in response to acute illness, which increases susceptibility to AKI [28].

A score equal to or above 2 in the HCT-CI was associated with a higher incidence of AKI in our study, in keeping with studies on AKI in allogeneic HSCT [11]. This comorbidity index has been validated for autologous HSCT [29] and we believe it should be taken into consideration as an important tool also for predicting AKI.

Chronic kidney disease was associated with an over twofold higher risk of developing AKI in our study, in line with the report of Andronesi et al. [13] in patients with autologous HSCT. In the last decade, CKD has been recognized as a risk factor for AKI [30].

Although our study only included 5 patients with concomitant amyloidosis, the presence of amyloidosis was associated with a higher risk of AKI. This was also suggested by Fadia et al. with a moderate-to-severe AKI incidence of 21% [24]. This may suggest that renal deposition of amyloid might reduce the renal ability to cope with nephrotoxic stimuli, even in the absence of overt CKD.  

Mucositis grade 3 and 4 was also associated with a higher risk of developing AKI. This finding was also reported by Andronesi et al [13] and Rodrigues et al [12] in autologous HSCT and is explained mainly by the extracellular volume depletion as a consequence of vomiting and diarrhea.

In our study, overall AKI was not associated with lower survival, unlike moderate to severe AKI (HR: 1.95; 95% CI 1.26–2.98) during the first 5 years after HSCT. Acute kidney injury in our study was not associated with disease-free survival, eGFR reduction, or chronic kidney disease progression.

The impact of AKI on overall survival was not consistently found in previous studies in patients with HSCT; however, a meta-analysis by Kanduri et al. [7] on the matter found an impact on mortality at 3 months and 3 years, higher in more severe stages, which is reinforced by our study with a longer follow up.

Before undergoing HSCT our patients had a median eGFR of 100.4 (89.5–110.8) mL/min/1.73 m^2^, and five years later, the survivors had a median eGFR of 82.6 (60.5–94.8) mL/min/1.73 m^2^, which represents a higher eGFR reduction than the 1 mL/min/m^2^ per year, usually retained as linked to aging, in the general population. The CKD prevalence before undergoing HSCT was 10.5% versus 27.5% at the end of the five-year follow-up, stressing the need for attention to, and early diagnosis of, CKD.

The retrospective nature is a limit of our study. Patients were often exposed to more than one nephrotoxic drug, which makes it difficult to identify the impact of each drug on AKI. Also, no data were available on structural abnormalities of the kidneys or proteinuria during follow-up to define CKD with more precision (besides the reduction of eGFR).

Still, to the best of our knowledge this is the first study using KDIGO classification with both creatinine criteria and urinary output criteria to analyze AKI incidence and main related factors in patients with multiple myeloma undergoing autologous HSCT. Prospective and multicenter studies are needed to study AKI in this growing population in order to provide better care to our patients.

## Data Availability

The data underlying this article will be shared on reasonable request to the corresponding author.
